# Manual of the Theory and Practice of Equine Medicine

**Published:** 1885-04

**Authors:** 


Manual of the Theory and Practice of Equine Medicine, by James Brodie Gresswell and Albert Gresswell. London: Balliére, Tindall & Cox; New York: William R. Jenkins. 1885.

The participation of two brothers—one of whom, a member of the Royal College of Veterinary Surgeons, is a practical veterinarian, while the other is a scholar of Christ Church, Oxford, a graduate "in honors" of the highest school of Physiology and Comparative Anatomy of the same school—would lead one to anticipate an excellent result from their combined labors. If this anticipation is not completely realized, it is due, we believe, rather to the limited size of the work than to any inability on the part of the authors; for what they have done in the three hundred and sixty-seven pages before us has been very well done as far as it goes. The style is clear, the statements brief, and the definitions concise and full. There is a little of everything in the book, and we can pay it no higher and better-deserved compliment than by saying that we only regret on behalf of those who studied veterinary medicine some years ago, that they had at that time no such useful and reliable a manual as this one to study as an introduction to the more thorough, but also more involved and voluminous treatises, which this little volume is not intended to replace, but merely to precede in the curriculum. There is an excellent frontispiece plate representing the *bacillus anthracis*.

